# Temporal heterogeneity in oxygen tension in human melanoma xenografts

**DOI:** 10.1038/sj.bjc.6601047

**Published:** 2003-07-15

**Authors:** K G Brurberg, B A Graff, E K Rofstad

**Affiliations:** 1 Group of Radiation Biology and Tumor Physiology, Department of Biophysics and Centre for Research and Training in Radiation Therapy, The Norwegian Radium Hospital, Montebello, N-0310 Oslo, Norway

**Keywords:** acute hypoxia, oxygen tension, *p*O_2_ fluctuations, temporal heterogeneity in *p*O_2_

## Abstract

The spatial heterogeneity of the oxygen tension (*p*O_2_) in human and experimental tumours has been studied extensively, whereas studies of the temporal heterogeneity in *p*O_2_ are sparse. In the work reported here, *p*O_2_ was measured continuously over periods of at least 60 min in A-07 human melanoma xenografts by using the OxyLite fibre-optic oxygen-sensing device. The main purpose of the work was to establish the usefulness of the OxyLite system in measuring the temporal heterogeneity in *p*O_2_ in tissues and to characterise the fluctuations in tissue *p*O_2_ in A-07 tumours. The OxyLite device was found to be suitable for studies of the temporal heterogeneity in *p*O_2_ in tumours. However, potential pitfalls were identified, and reliable *p*O_2_ measurements require that precautions are taken to avoid these pitfalls, that is, erroneous *p*O_2_ readings caused by tissue trauma induced by the probe, probe movements induced by reflex actions of the host mouse and occasional probe drift. Significant fluctuations in *p*O_2_ were detected in the majority of the 70 tumour regions subjected to measurement. The fluctuations in different regions of the same tumour were in general temporally independent, implying that they were caused primarily by redistribution of the tumour perfusion rather than fluctuations in global perfusion. Fourier analysis of the *p*O_2_ traces showed that the *p*O_2_ usually fluctuated at frequencies lower than 1.5–2.0 mHz, corresponding to less than 0.1 cycle min^−1^. Haemodynamic effects may cause *p*O_2_ fluctuations in this frequency range, and hence, the redistribution of the perfusion could have been caused by morphological abnormalities of the tumour microvasculature. Moreover, acute hypoxia, that is, *p*O_2_ fluctuations around 10 or 5 mmHg, was detected in 20 of 70 regions, that is, 29% (10 mmHg), or 27 of 70 regions, that is, 39% (5 mmHg). The median fraction of the time these regions were acutely hypoxic was 73% (10 mmHg) or 53% (5 mmHg). Consequently, if A-07 tumours are adequate models of tumours in man, acute hypoxia may be a commonly occurring phenomenon in neoplastic tissues, and hence, acute hypoxia is likely to cause resistance to radiation therapy and promote tumour aggressiveness.

Most tumours are heterogeneous in oxygen tension (*p*O_2_) and show regions with severely hypoxic cells (Vaupel *et al*, 1989). Two main types of hypoxia have been recognised: chronic hypoxia, arising from limitations in oxygen diffusion, and acute hypoxia, resulting from transient cessations in microregional blood flow (Horsman, 1995; Brown, 1999). Tumour hypoxia causes resistance to radiation therapy and some forms of chemotherapy (Brown, 1999; Wouters *et al*, 2002) and promotes malignant progression and the development of metastatic disease (Rofstad, 2000; Höckel and Vaupel, 2001). Clinical investigations have shown that extensive hypoxia in the primary tumour is associated with locoregional treatment failure and poor disease-free and overall survival in several histological types of cancer (Rofstad, 2000; Höckel and Vaupel, 2001; Wouters *et al*, 2002).

The spatial heterogeneity in *p*O_2_ in tumours has been studied in great detail (Vaupel *et al*, 1989; Vaupel, 1990). Steep gradients in *p*O_2_ and *p*O_2_ values ranging from 0 mmHg to those found in well-oxygenated normal tissues are characteristic features of tumours, implying that tumour tissues may show extensive spatial heterogeneity in cellular radiation sensitivity (Wouters and Brown, 1997) and hypoxia-induced gene expression (Acker and Plate, 2002; Harris, 2002). In contrast, studies of the temporal heterogeneity in *p*O_2_ in tumours are sparse (Dewhirst *et al*, 2000). However, examinations of fluctuations in microvessel blood flow in window chamber tumours and measurements of the kinetics of red blood cell flux in experimental and human solid tumours by laser Doppler flowmetry have suggested that temporal heterogeneity in *p*O_2_ and acute hypoxia are common events in tumour tissues (Endrich *et al*, 1979; Brizel *et al*, 1993; Chaplin and Hill, 1995; Hill *et al*, 1996). Moreover, radiobiological studies of murine tumours and human tumour xenografts have given evidence that acute hypoxia may be a significant cause of tumour radiation resistance (Yamaura and Matsuzawa, 1979; Chaplin *et al*, 1987; Rofstad and Måseide, 1999). Measurements of temporal heterogeneity in *p*O_2_, however, have been reported for tumours of one line only, the rat R3230Ac mammary adenocarcinoma. In these experiments, recessed-tip oxygen microelectrodes (Linsenmeier and Yancey, 1987) were used to record fluctuations in *p*O_2_ in tumours implanted subcutaneously (Dewhirst *et al*, 1998; Braun *et al*, 1999) or in window chambers (Dewhirst *et al*, 1996; Kimura *et al*, 1996). The studies suggested that tissue *p*O_2_ and red blood cell flux fluctuated at low frequencies and were temporally coordinated, and demonstrated that acute hypoxia is a frequently occurring phenomenon in R3230Ac tumours (Dewhirst *et al*, 1996,^1998^; Kimura *et al*, 1996; Braun *et al*, 1999).

The low number of investigations of the temporal heterogeneity in *p*O_2_ in tumours most likely reflects that adequate equipment for measurement of fluctuations in *p*O_2_ in tissues has not been commercially available (Dewhirst *et al*, 2000). A new oxygen-sensing device, the OxyLite system, was marketed recently (Young *et al*, 1996; Griffiths and Robinson, 1999). This device measures *p*O_2_ by using a fluorescence quenching technique. Light pulses induce fluorescence in ruthenium chloride incorporated into a silicone rubber polymer at the tip of 220-*μ*m-diameter fibre-optic probes. The lifetime of the fluorescence is inversely proportional to the oxygen tension at the probe tip. Comparative studies have shown that tumour *p*O_2_ distributions and hypoxic fractions measured with the OxyLite system are similar to those obtained with Eppendorf polarographic electrodes (Collingridge *et al*, 1997; Seddon *et al*, 2001), recessed-tip microelectrodes (Braun *et al*, 2001) and radiobiological assays (Urano *et al*, 2002). The OxyLite system has been used successfully to measure *p*O_2_ changes in experimental tumours following different types of physiological intervention (Bussink *et al*, 2000; Braun *et al*, 2001;Demeure *et al*, 2002; Jarm *et al*, 2002; Jordan *et al*, 2002).

In the work reported here, the OxyLite system was used to measure *p*O_2_ fluctuations in unperturbed A-07 human melanoma xenografts transplanted orthotopically in BALB/c-*nu/nu* mice. Xenografted A-07 tumours have been shown to retain many biological characteristics of the donor patient's tumour, including cell cycle distribution, angiogenic potential, vascular density, metastatic pattern and several histological and pathophysiological parameters (Rofstad, 1994). The main purpose of the work was to establish the usefulness of the OxyLite system in studying the temporal heterogeneity in *p*O_2_ in tissues and to characterise the *p*O_2_ fluctuations in a clinically relevant tumour model. Significant evidence was found that the *p*O_2_ in many tumour regions fluctuated around the threshold values for hypoxia-induced radiation resistance and hypoxia-induced gene expression.

## MATERIALS AND METHODS

### Mice and tumours

Adult (8−10 weeks of age) female BALB/c-*nu/nu* mice were used as host animals for xenografted tumours. The mice were maintained under specific pathogen-free conditions at constant temperature (37.0±0.5°C) and humidity (50−60%). Sterilised food and water were given *ad libitum*. Experiments were performed with tumours of the A-07 human melanoma line, established as described previously (Rofstad, 1994). Tumours were initiated from exponentially growing cell cultures verified to be free from *Mycoplasma* contamination. The cells were cultured in RPMI-1640 medium (25 mM HEPES and L-glutamine) supplemented with 13% bovine calf serum, 250 mg l^−1^ penicillin and 50 mg l^−1^ streptomycin. Approximately 3.5 × 10^5^ cells in 10 *μ*l of Ca^2+^ and Mg^2+^-free Hanks' balanced salt solution were inoculated intradermally into the hindmost part of the left mouse flank by using a 100-*μ*l Hamilton syringe. The tumours were subjected to measurements of *p*O_2_ when having volumes within the range of 300−700 mm^3^. Tumour volume (*V*) was calculated as *V*=(*π*/6)*ab*^2^, where *a* is the longer and *b* is the shorter of two perpendicular diameters, measured with calipers. Animal experiments were approved by the Institutional Committee on Research Animal Care and were performed in accordance with the ethical standards of the UKCCCR ‘Guidelines for the Welfare of Animals in Experimental Neoplasia’ (Workman *et al*, 1998).

### Measurement of *p*O_2_

Tumour *p*O_2_ was measured with a two-channel fibre-optic oxygen-sensing device (OxyLite™ 2000, Oxford Optronix, Oxford, UK). The probes were supplied precalibrated by the manufacturer. The calibration data were scanned into a computer by means of a barcode wand. The calibration specification was ±0.7 mmHg or <±10% of actual *p*O_2_, whichever was greater. To ensure correct *p*O_2_ readings, the accuracy of the precalibration data was controlled for every probe by measuring *p*O_2_ in Ringers solutions flushed with N_2_ gas containing 0, 0.5, 1.0, 2.5 or 5.0% O_2_. This procedure was repeated at the end of each experiment to control for drift in the system. Atmospheric pressure was measured with a DPI 705 barometer (Newport Electronics, Deckenpfronn, Germany).

The mice were kept under anaesthesia during the *p*O_2_ measurements. A mixture of fentanyl citrate/fluanisone (Janssen Pharmaceutika, Beerse, Belgium) and midazolam (Roche, Basel, Switzerland) was administered intraperitoneally in doses of 0.63/20 and 10 mg kg^−1^, respectively. The body core temperature of the mice, measured with a rectal probe, was kept at 37.5–38.0°C by using a heating lamp and a heating pad. The tumour temperature under these conditions, measured with an OxyLite probe having a fine thermocouple wound around the optic fibre, was within 37.0–38.0°C, that is, it was similar to the tumour temperature during the growth period prior to the *p*O_2_ measurements.

Most tumours had an ellipsoidic shape, and tissue *p*O_2_ was measured simultaneously in two positions in each tumour, that is, centrally in each of the two halves of the ellipsoid. The probes were inserted into the tumours through skin punctures made with 21- or 23-gauge needles. After insertion, the probes were retracted by approximately 1 mm to minimise the pressure on the sensors before they were fixed firmly and *p*O_2_ measurements were initiated. The *p*O_2_ readings were recorded every 5, 10 or 30 s for at least 60 min and were stored to disc and displayed as time traces by using a data-acquisition system (LabView, National Instruments, Austin, TX, USA). The mice were watched continuously during the experiments and reflex movements were recorded. At the end of the experiments, the mice were killed without withdrawing the probes from the tumour tissue, and it was ensured that the *p*O_2_ dropped rapidly to 0 mmHg. Data were discarded if (a) changes in tumour *p*O_2_ were associated with reflex movements of the host mouse, (b) tumour *p*O_2_ did not drop to 0 mmHg after killing of the host mouse or (c) the probe calibration at the end of an experiment gave data that differed significantly from the precalibration data.

### Fourier analysis

Characteristic frequencies of *p*O_2_ fluctuations were searched for by subjecting the *p*O_2_ traces (oxygen tension *vs* time) to Fourier analysis. The Fourier analysis was performed by using IDL software (Research Systems, Boulder, CO, USA). The program calculated frequency spectra from *p*O_2_ traces by accomplishing a discrete Fourier transformation. The highest detectable frequency was given by the sampling frequency and was 100 mHz at a sampling frequency of 5 s. The lowest detectable frequency was given by the length of the observation period and was approximately 0.4 mHz at an observation period of 40 min.

## RESULTS

**Figure 1:**
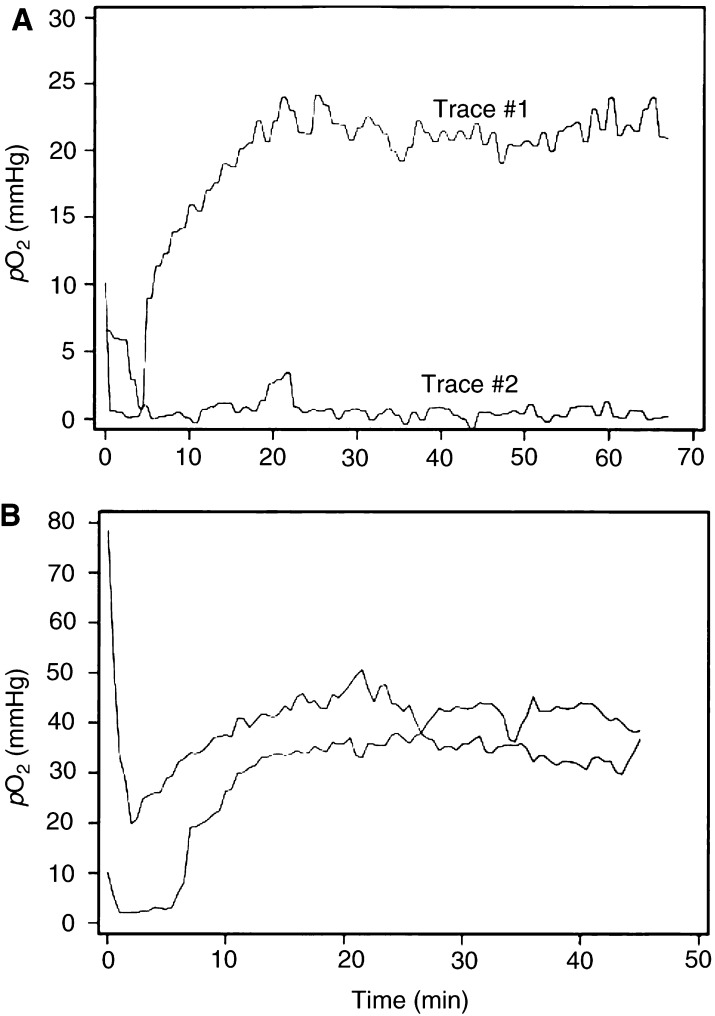
Examples of *p*O_2_ traces recorded with OxyLite probes in A-07 tumours (**A**) and muscle tissue (**B**). The traces show that the changes in *p*O_2_ were more pronounced shortly after the probe insertion than towards the end of the observation period.

Measurement of *p*O_2_ in A-07 tumours with OxyLite probes gave values that varied substantially with time. The variations were more pronounced shortly after the probe insertion than towards the end of the observation period. Two characteristic *p*O_2_ traces are illustrated in Figure 1A

. The initial *p*O_2_ was above 5 mmHg in both traces. In trace #1, the *p*O_2_ decreased for several minutes before it increased gradually and reached a high and fairly stable level. In trace #2, the *p*O_2_ decreased rapidly with time before a low and fairly stable level was reached. To investigate whether the initial changes in *p*O_2_ were artefacts caused by the probe insertion, similar *p*O_2_ measurements were performed in muscle tissue in BALB/c-*nu/nu* mice. Two typical *p*O_2_ traces from muscle tissue are presented in Figure 1B. The muscle *p*O_2_ traces were qualitatively similar to *p*O_2_ trace #1 in Figure 1A, strongly suggesting that the initial *p*O_2_ changes were artefacts.

**Figure 2:**
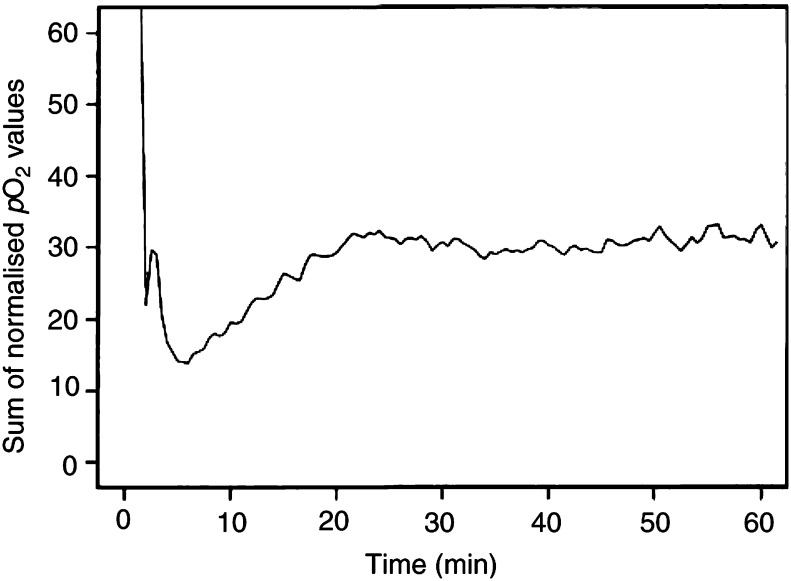
Sum of 50 normalised *p*O_2_ traces recorded with OxyLite probes in A-07 tumours. The composed trace shows that the changes in *p*O_2_ recorded within the first 20 min after the probe insertion were systematic, whereas those recorded beyond the first 20 min were random.

To determine the length of the time period in which the *p*O_2_ readings in A-07 tumours obviously were influenced by the probe insertion, 50 randomly selected *p*O_2_ traces were normalised and summed. The normalisation was performed by dividing all *p*O_2_ values in a trace by the highest *p*O_2_ value recorded in that trace, excluding *p*O_2_ values recorded during the first 10 min when determining the normalisation factor. The *p*O_2_ trace representing the sum of the normalised *p*O_2_ traces is plotted in Figure 2

. This plot suggests that the changes in *p*O_2_ recorded within the first 20 min after the probe insertion were systematic and hence were artefacts caused by the probe, whereas the *p*O_2_ changes recorded beyond 20 min were random and hence most likely represented true variations in tissue *p*O_2_. Consequently, the temporal heterogeneity in *p*O_2_ in A-07 tumours was studied by only considering *p*O_2_ values recorded beyond the first 20 min after the insertion of a probe, that is, the first 20 min of each *p*O_2_ trace was excluded in the analysis presented below.

**Figure 3:**
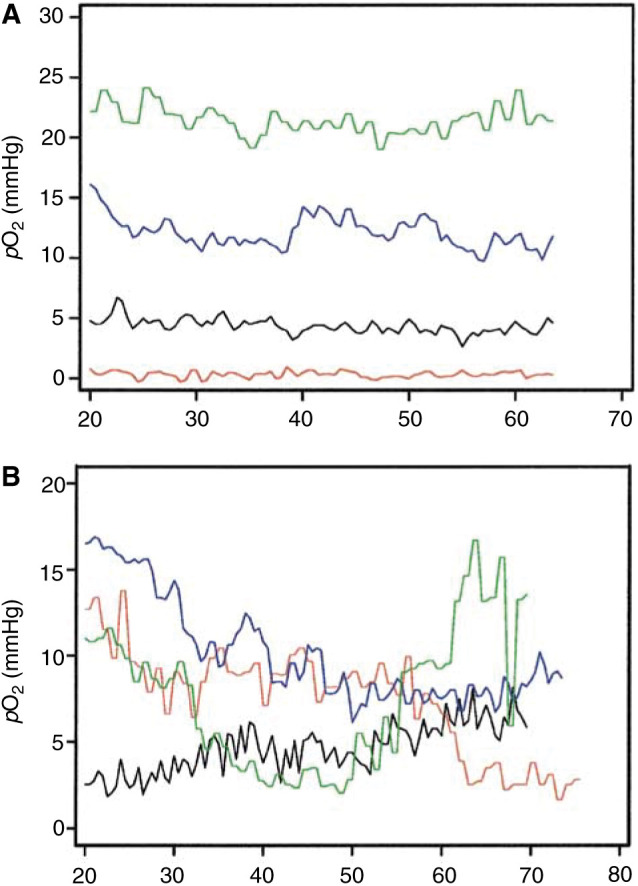
Examples of *p*O_2_ traces recorded with OxyLite probes in A-07 tumours. The traces refer to tumour regions without significant fluctuations in *p*O_2_ (**A**) and tumour regions showing significant fluctuations in *p*O_2_ around *p*O_2_ values of 5 and 10 mmHg (**B**).

A total of 38 A-07 tumours were subjected to *p*O_2_ measurements and a total of 70 reliable *p*O_2_ traces were obtained. The *p*O_2_ traces showed substantial differences, regardless of whether they were recorded in the same tumour or in different tumours. Mean *p*O_2_ differed among the traces from 0 to 38 mmHg. Examples of characteristic *p*O_2_ traces are illustrated in Figure 3

. Both well-oxygenated and poorly oxygenated tumour regions could show *p*O_2_ traces without significant fluctuations (Figure 3A). However, significant *p*O_2_ fluctuations were detected in most tumour regions (Figure 3B). The *p*O_2_ traces were analysed by using two threshold values for hypoxia, that is, *p*O_2_=5 and 10 mmHg. Some tumour regions were not hypoxic at all during the observation period, whereas others were hypoxic during the entire period (Table 1

**Table 1:** Parameters describing the kinetics of acute hypoxia in A-07 human melanoma xenografts and R3230Ac rat mammary adenocarcinomas

	**A-07**		**R3230Ac^a^**	
**Parameter**	***p*O_2_<10 mmHg**	***p*O_2_<5 mmHg**	***p*O_2_<10 mmHg**	***p*O_2_<5 mmHg**
Tumour regions that were never hypoxic (%)	17 (12/70)	30 (21/70)	15 (2/13)	38 (5/13)
Tumour regions that were always hypoxic (%)	54 (38/70)	31 (22/70)	23 (3/13)	23 (3/13)
Tumour regions showing acute hypoxia (%)	29 (20/70)	39 (27/70)	62 (8/13)	38 (5/13)
Hypoxic periods per *p*O_2_ trace per hour (# h^−1^)	2.7 (1.1–8.5)^b^	4.3 (1.1–12.0)	6.7 (2.7–24.0)	3.9 (0.9–17.9)
Fractional time of hypoxia per *p*O_2_ trace (%)	73.0 (1.9–99.5)	53.4 (4.3–97.9)	59.5 (30.8–90.0)	60.1 (16.2–84.7)
Duration of hypoxic periods (min)	4.6 (0.5–49.5)	2.0 (0.5–44.0)	5.0 (1.2–16.6)	7.4 (0.9–44.7)

a Data from Dewhirst *et al* (1998).

b Median value and range.

). Acute hypoxia, that is, *p*O_2_ fluctuations around the threshold values, was detected in 29% (10 mmHg) and 39% (5 mmHg) of the tumour regions. To characterise the kinetics of the acute hypoxia, the number of times per hour the *p*O_2_ decreased below the threshold values and the fractional time the *p*O_2_ was below the threshold values were calculated for each of the tumour regions showing acute hypoxia. The durations of the hypoxic periods were also determined. The median values and the ranges of these parameters are presented in Table 1. A similar analysis has been performed previously for R3230Ac rat tumours (Dewhirst *et al*, 1998), and the results of this analysis were included in Table 1 for comparison.

**Figure 4:**
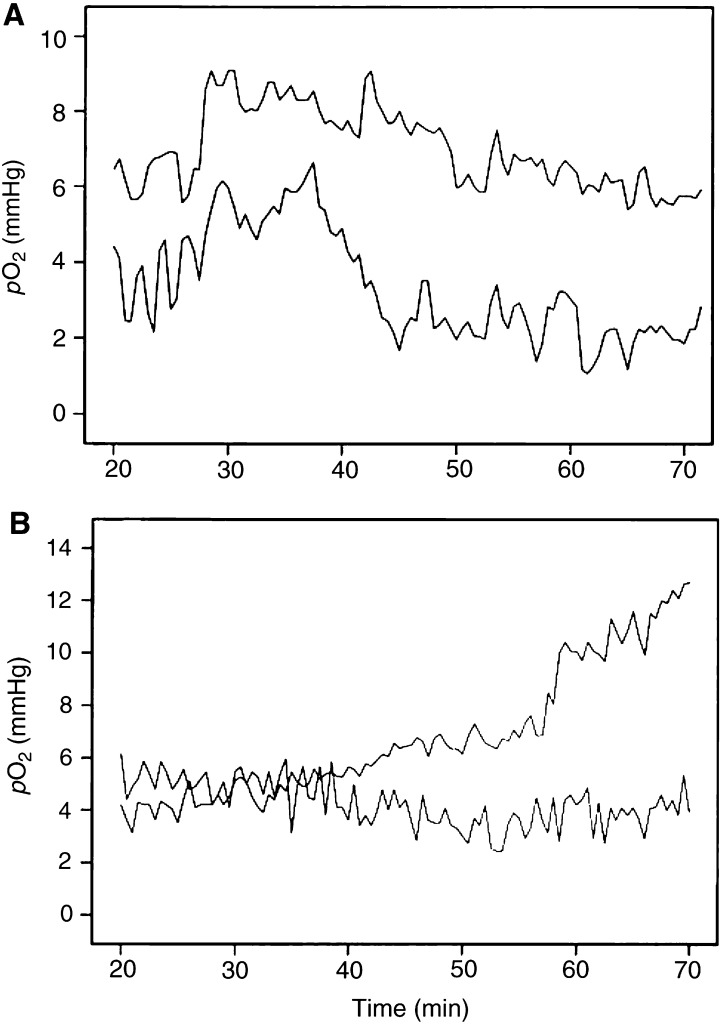
Examples of *p*O_2_ traces recorded simultaneously with OxyLite probes in two regions of the same A-07 tumours. The traces refer to a tumour where the *p*O_2_ values were correlated (**A**) and a tumour where the *p*O_2_ values were inversely correlated (**B**).

Two *p*O_2_ traces were recorded simultaneously in most tumours. None of the traces, one of the traces, or both traces could show significant *p*O_2_ fluctuations. The *p*O_2_ values of concurrent traces were subjected to correlation analysis to investigate whether the *p*O_2_ fluctuations in different regions of the same tumour were temporally coordinated. Positive correlations were found in some tumours (Figure 4A

), whereas inverse correlations were seen in others (Figure 4B). However, there was no correlation between the two series of *p*O_2_ values in the majority of the tumours, implying that the *p*O_2_ fluctuations in different regions of a tumour in general were temporally independent.

**Figure 5:**
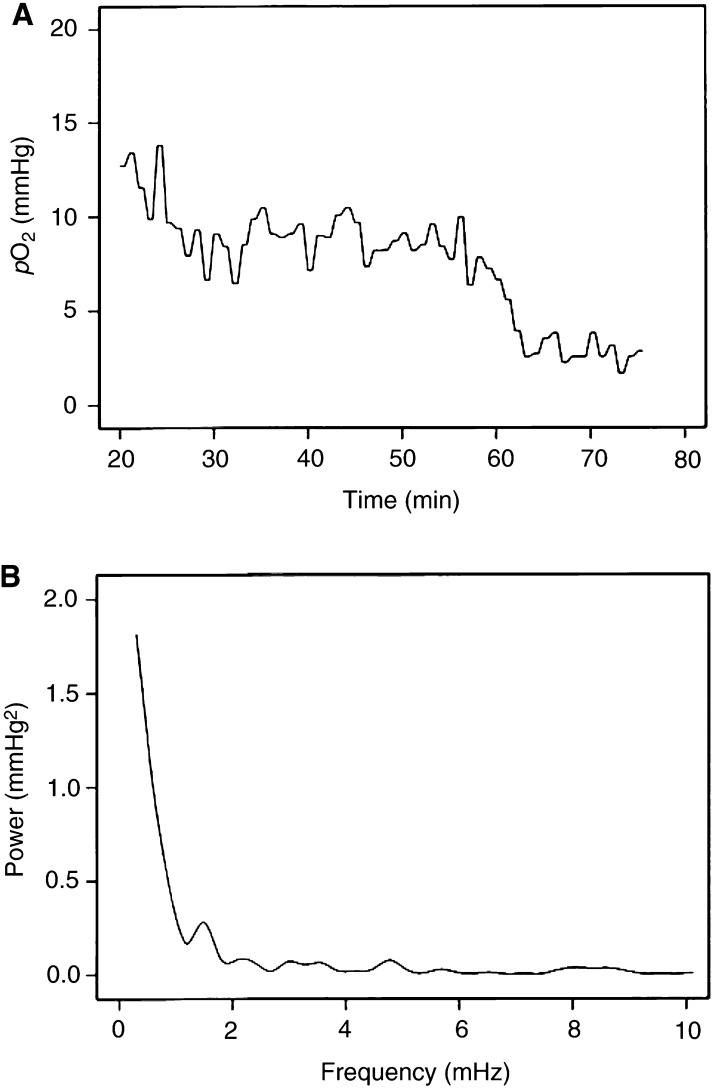
Example of a *p*O_2_ trace recorded with an OxyLite probe in an A-07 tumour (**A**) and the corresponding frequency spectrum (**B**). The frequency spectrum, which was obtained by subjecting the *p*O_2_ data to Fourier analysis, suggests that the *p*O_2_ fluctuated at frequencies lower than 1.5–2.0 mHz.

Moreover, the *p*O_2_ traces were subjected to Fourier analysis to investigate whether the *p*O_2_ fluctuated at characteristic frequencies. The Fourier analysis resulted in frequency spectra that were not qualitatively different, regardless of whether they were derived from the same tumour or from different tumours. The frequency spectra indicated that the *p*O_2_ fluctuated at very low frequencies, that is, at frequencies lower than 1.5–2.0 mHz, corresponding to less than 0.1 cycle min^−1^. Significant fluctuations at higher frequencies could not be detected. Data from a characteristic tumour region are presented in Figure 5

, showing the *p*O_2_ trace (Figure 5A) and the corresponding frequency spectrum (Figure 5B).

## DISCUSSION

Tissue *p*O_2_ in A-07 human melanoma xenografts was monitored continuously over periods of at least 60 min by using OxyLite fibre-optic probes. The study showed that fluctuations in *p*O_2_ at the microregional level occur frequently in A-07 tumours. Moreover, acute hypoxia was found to be a common phenomenon in these tumours, which is in agreement with the conclusions from a previous study in which radiobiological and immunohistochemical assays were used to detect hypoxia in A-07 and other human melanoma xenografts (Rofstad and Måseide, 1999). The OxyLite system has been used previously to study changes in tumour *p*O_2_ following treatment with blood flow and tissue oxygenation modifying agents (Bussink *et al*, 2000; Braun *et al*, 2001;Demeure *et al*, 2002; Jarm *et al*, 2002; Jordan *et al*, 2002). The present study is the first in which the OxyLite system has been used successfully to study temporal heterogeneity in *p*O_2_ in unperturbed tumours.

Studies of temporal heterogeneity in *p*O_2_ in tumour tissue with the OxyLite system, however, require precautions against potential methodical pitfalls, as revealed by the work reported here. First, it was observed that the *p*O_2_ values recorded shortly after the probe was inserted into tumour tissue varied systematically with time and hence were influenced signifcantly by the probe insertion. A similar artefact was seen when tissue *p*O_2_ was measured in P22 rat carcinosarcomas with OxyLite probes (Seddon *et al*, 2001). The *p*O_2_ readings stabilised after approximately 10 min in P22 tumours, whereas in A-07 tumours, reliable *p*O_2_ readings could not be obtained until 20 min after the probe insertion. The artificial *p*O_2_ readings obtained during the first 20 min of measurement in A-07 tumours were most likely a result of vasoconstrictive reactions to tissue trauma induced by the probe. However, other factors may also have contributed, as discussed in detail for P22 tumours (Seddon *et al*, 2001). Second, it was observed that abrupt changes in the *p*O_2_ readings could occur simultaneously with reflex movements of the host mouse. These changes did probably not reflect temporal heterogeneity in *p*O_2_, but were rather a consequence of minor changes in probe position and hence the spatial heterogeneity in *p*O_2_, as it is well known that tumour tissues can show steep *p*O_2_ gradients (Vaupel, 1990; Horsman, 1995; Braun *et al*, 2001; Urano *et al*, 2002). Third, it was observed that some correctly precalibrated probes after a few measurements in tissue suddenly began recording erroneous absolute values of *p*O_2_. Therefore, it is essential to kill the host animals after each experiment and ensure that the *p*O_2_ drops rapidly to 0 mmHg, and to control the probe calibration regularly in Ringers solutions, as was followed in the work reported here.

Our analysis was based on the assumption that the *p*O_2_ readings recorded beyond the first 20 min after the probe insertion were not influenced significantly by the tissue trauma caused by the probe. The OxyLite probes have a tip diameter of 220 *μ*m and may therefore now and then cause severe tissue damage during the insertion, for example by destroying or compressing larger vessels. Consequently, it cannot be excluded that the *p*O_2_ readings in some of the tumour regions studied here were influenced by probe-induced tissue damage also beyond the first 20 min. However, several observations suggest that this potential problem, if present, was of minor significance. First, *p*O_2_ was measured in normal tissues also, and significant *p*O_2_ fluctuations were never observed beyond 20 min, as illustrated for muscle tissue in Figure 1B. Second, we have shown that the mean *p*O_2_ measured beyond 20 min is inversely correlated to the fraction of radiobiologically hypoxic cells in A-07 and R-18 human melanoma xenografts (Brurberg *et al*, unpublished data). Moreover, comparative studies performed in other laboratories have demonstrated that the *p*O_2_ distributions measured in experimental tumours with OxyLite probes are similar to those obtained with Eppendorf polarographic electrodes (Collingridge *et al*, 1997; Seddon *et al*, 2001) and recessed-tip microelectrodes (Braun *et al*, 2001).

Previous studies of the rat R3230Ac mammary adenocarcinoma have led to the suggestion that fluctuations in tissue *p*O_2_ and acute hypoxia may be commonly occurring phenomena in tumours (Dewhirst *et al*, 1996,^1998^; Kimura *et al*, 1996; Braun *et al*, 1999). The present study of A-07 human melanoma xenografts, which have been shown to retain several characteristic biological features of the donor patient's tumour (Rofstad, 1994) and hence most likely are more relevant models of tumours in man than are R3230Ac tumours, confirmed this suggestion. A direct comparison of the temporal heterogeneity in *p*O_2_ in A-07 and R3230Ac tumours is difficult, however, because OxyLite fibre-optic probes were used to measure *p*O_2_ in the present work and recessed-tip microelectrodes were used to measure *p*O_2_ in the R3230Ac tumours. The tip diameter of the OxyLite probes is 220 *μ*m, and the sampling volume has been estimated to be approximately 1000 cells (Griffiths and Robinson, 1999; Seddon *et al*, 2001). In contrast, the recessed-tip microelectrodes had a diameter of only 10–12 *μ*m (Dewhirst *et al*, 1998), and therefore, they had a sampling volume that was substantially smaller than that of the OxyLite probes (Braun *et al*, 2001). Since tumours are spatially heterogeneous in *p*O_2_ at the microregional level (Vaupel, 1990; Horsman, 1995), microelectrodes are expected to measure larger fluctuations in *p*O_2_ in tumours than are OxyLite probes (Braun *et al*, 2001). Nevertheless, the temporal heterogeneity in *p*O_2_ measured in A-07 tumours was remarkably similar to that measured in R3230Ac tumours, as can be seen from the comparison of A-07 and R3230Ac tumours presented in Table 1.

Temporal heterogeneity in *p*O_2_ in tumour tissue has to be caused by temporal heterogeneity in either oxygen delivery, that is, blood supply, or oxygen consumption, that is, cell respiration, or both. There is no experimental evidence that the rate of respiration may fluctuate synchronously in cells within tumour microregions. On the other hand, there is ample evidence that the blood supply may fluctuate significantly at the microregional level in both experimental and human tumours (Endrich *et al*, 1979; Brizel *et al*, 1993; Chaplin and Hill, 1995; Hill *et al*, 1996). Studies of R3230Ac tumours transplanted to window chambers have suggested that the fluctuations in *p*O_2_ in these tumours were temporally coordinated with fluctuations in red blood cell flux (Dewhirst *et al*, 1996; Kimura *et al*, 1996). Moreover, Fourier analysis of *p*O_2_ traces recorded with recessed-tip microelectrodes and red blood cell flux traces recorded by laser Doppler flowmetry revealed that both *p*O_2_ and blood flow fluctuated at very low frequencies in subcutaneous R3230Ac tumours (Braun *et al*, 1999). The data reported here for A-07 tumours are in good agreement with those of the R3230Ac tumours. Thus, the *p*O_2_ frequency spectra of the A-07 tumours suggested that the *p*O_2_ fluctuated at low frequencies also in these tumours, that is, at frequencies lower than 1.5–2.0 mHz, corresponding to less than 0.1 cycle min^−1^. Fluctuations in *p*O_2_ in this frequency range could result from fluctuations in blood flow caused by vasomotion in supplying arterioles, haemodynamic mechnisms and/or microvascular remodelling via intussusceptive vascular growth (Braun *et al*, 1999). Haemodynamic mechanisms may be particularly significant because the blood viscosity is elevated in tumour tissues and the tumour microvascular network is irregular and chaotic (Vaupel *et al*, 1989).

Studies of intratumour heterogeneity in temporal variation in *p*O_2_ have not been reported so far. The *p*O_2_ measurements performed in R3230Ac tumours were all restricted to a single point at a time in each tumour (Dewhirst *et al*, 1996,^1998^; Kimura *et al*, 1996; Braun *et al*, 1999). Therefore, these studies did not provide information on the fractional tumour volume showing *p*O_2_ fluctuations or on the temporal coordination of the *p*O_2_ fluctuations in different tumour regions. Attempts to obtain information of this type were made in the present work by measuring *p*O_2_ simultaneously in two distinctly different regions of the same A-07 tumours. In some tumours, significant fluctuations in *p*O_2_ could not be detected in any of the regions, and in others, *p*O_2_ fluctuated significantly in one of the regions only, suggesting that the *p*O_2_ fluctuations usually involved only a fraction of the tumour volume. Many A-07 tumours, however, showed significant *p*O_2_ fluctuations in both regions. These fluctuations were in general not temporally coordinated, suggesting that they were caused primarily by redistribution of the perfusion within the tumours rather than changes in global perfusion. Thus, in some tumours, the *p*O_2_ values in the two regions analysed simultaneously were inversely correlated. However, our experiments can by no means exclude the possibility that also the global perfusion and hence the fraction of acutely hypoxic cells varied significantly with time. In fact, this possibility is very likely, considering the irregular and heterogeneous nature of the microvasculature of tumours, and is supported by the observation that the two *p*O_2_ series recorded simultaneously were strongly correlated in some tumours. Studies of temporal heterogeneity in *p*O_2_ involving mapping of the *p*O_2_ distribution in whole tumours are needed, however, before this question can be settled.

The study reported here may have significant implications for the radiation therapy of cancer. First, clinical studies attempting to eliminate the chronic hypoxia in tumours during radiation therapy are being performed, but so far, the therapeutic results have been unsatisfactory (Overgaard and Horsman, 1996). The present observations suggest that a significant fraction of the hypoxic cells in tumours are acutely hypoxic, and acutely hypoxic cells may be more resistant to radiation therapy than chronically hypoxic cells (Pettersen and Wang, 1996; Zölzer and Streffer, 2002). Consequently, treatment strategies aiming at reducing the fraction of acutely hypoxic cells may prove more successful in improving the outcome of radiation therapy than those aiming at reducing the fraction of chronically hypoxic cells. Second, serious attempts have been initiated to improve the local control of radiation-resistant tumours by using intensity modulated radiation therapy for selective boosting of hypoxic subvolumes (Tome and Fowler, 2000; Chao *et al*, 2001; Popple *et al*, 2002). Our observations suggest that the spatial distribution of the acutely hypoxic regions in tumours may change rapidly with time. Consequently, efficient selective boosting of hypoxic subvolumes may require novel technology for imaging of tumour hypoxia and guiding of intensity-modulated radiation therapy.

The present observations are also relevant for our understanding of the malignant progression of tumours and the development of metastatic disease. It is well established that tumour hypoxia activates DNA transcription factors, for example, HIF-1, and leads to increased expression of a large number of genes, and a high expression of these genes has been shown to be associated with poor prognosis in several histological types of cancer (Rofstad, 2000; Höckel and Vaupel, 2001; Acker and Plate, 2002; Harris, 2002; Wouters *et al*, 2002). Some of the genes that are activated under hypoxic conditions encode proteins involved in the metastatic process, for example, angiogenesis factors and proteolytic enzymes. Studies of human melanoma xenografts have shown that tumour hypoxia may promote metastasis by upregulating the expression of interleukin-8 (Rofstad and Halsør, 2002) and urokinase-type plasminogen activator receptor (Rofstad *et al*, 2002). The study reported here showed that a significant fraction of the hypoxic volume of tumours may be acutely hypoxic and that the spatial distribution of the acutely hypoxic regions may change rapidly with time. It is possible that the dynamic nature of the acute hypoxia in tumours may lead to hypoxia-induced gene expression without loss of viability in a substantial fraction of the malignant cells and hence may promote aggressiveness and metastatic spread. Consistent with this suggestion is the observation that experimentally imposed acute hypoxia but not chronic hypoxia-enhanced spontaneous metastasis in KHT murine tumours (Cairns *et al*, 2001).

In summary, the present study showed that significant fluctuations in tissue *p*O_2_ and acute hypoxia are commonly occurring phenomena in A-07 human melanoma xenografts. If A-07 tumours are relevant models of tumours in man, acute hypoxia may be an important cause of resistance to radiation therapy and malignant progression in human cancer.
